# Enabling the flow of compassionate care: a grounded theory study

**DOI:** 10.1186/s12913-017-2120-8

**Published:** 2017-03-03

**Authors:** Stephanie Tierney, Kate Seers, Elizabeth Tutton, Joanne Reeve

**Affiliations:** 1 Royal College of Nursing Research Institute, Warwick Medical School, University of Warwick, Coventry, CV4 7AL UK; 2 Social Science and Systems in Health, Warwick Medical School, University of Warwick, Coventry, UK; 3 Trauma Research, Kadoorie Centre, Oxford University Hospitals NHS Foundation Trust, Coventry, UK

**Keywords:** Compassionate care, Qualitative research, Grounded theory, Type 2 diabetes, Interviews, Focus groups, Healthcare professionals

## Abstract

**Background:**

Compassion has become a topic of increasing interest within healthcare over recent years. Yet despite its raised profile, little research has investigated how compassionate care is enacted and what it means to healthcare professionals (HCPs). In a grounded theory study, we aimed to explore this topic from the perspective of people working with patients with type 2 diabetes – a long-term condition that involves repeated interactions with HCPs.

**Methods:**

Semi-structured interviews and focus groups were conducted between May and October 2015 with 36 participants, selected from a range of roles within healthcare. Data collection explored their understanding of compassionate care and experiences of it in practice. Analysis followed the constructivist approach of Charmaz, which recognises meaning as being created by the interaction of people working under specific sociocultural conditions. It moved from open to focused coding, and involved the development of memos and constant comparison.

**Results:**

Our analysis revealed that wishing to provide compassionate care, on its own, was insufficient to ensure this transpired; HCPs needed to work in a setting that supported them to do this, which underpins our core concept - the compassionate care flow. Data suggested that to be sustained, this flow was energised via what participants described as ‘professional’ compassion, which was associated with the intention to improve patient health and participants’ role within healthcare. The compassionate care flow could be enhanced by defenders (e.g. supportive colleagues, seeing the patient as a person, drawing on their faith) or depleted by drainers (i.e. competing demands on time and resources), through their impact on professional compassion.

**Conclusions:**

This paper presents a model of compassionate care based on the notion of flow. It looks at processes associated with this concept and how compassionate care is delivered within health settings. Our new understanding of this phenomenon will help those working in healthcare, including managers and policy makers, to consider and potentially offset disruption to the compassionate care flow.

## Background

Compassion is regarded commonly as a defining aspect of healthcare. For example, in the UK it is included as a value in the National Health Service Constitution [1], and it is part of the American Medical Association’s Principles of Medical Ethics [2]. In recent years, compassion within health settings has received increased attention, fuelled by concerns that it may be diminishing following high profile reports of poor patient care [3, 4]. As will be outlined below, compassion can be considered from a variety of perspectives. However, a lack of clarity around how to define compassion in healthcare specifically can mean that attempts to ensure it occurs in practice are impeded [5]. Research presented in this paper goes some way to addressing this gap in understanding by exploring how healthcare professionals (HCPs) make sense of compassion within their workplace when supporting people who have a long-term condition, type 2 diabetes.

Compassion is evident in literature within science and the humanities. It is said to have an evolutionary basis, marking a point in human development when we could think about others’ feelings [6]. As a consequence, humans were able to care for those in need and to exhibit concern for them [7]. This underlines the relational aspect of compassion [5, 8, 9], which necessitates an understanding of and connection with another [5, 10]. Compassion is also referenced in religious texts and belief systems [11], including Buddhism, where it constitutes being open and having an active response to suffering [12], guided by “reason and wisdom which is embedded in an ethical framework concerned with the selfless intention of freeing others from suffering” [11]. Compassion, therefore, connotes a deep connection with someone’s suffering [13], accompanied by the intention to act to alleviate this situation [9].

Suffering itself has been equated with real or anticipated loss [14], on a physical, social, cultural or spiritual level [15]. It is defined as “an affliction of the person, not the body…a specific state of distress that occurs when the intactness or integrity of the person is threatened or disrupted” [16]. In healthcare, suffering may take a range of forms, including physical discomfort, with symptom management necessary to enable patients to focus on other areas of life creating distress (e.g. loneliness, confusion, shame, guilt) [17]. Consequently, controlling pain may be required alongside attending to patients’ vulnerability and fears of what the future holds, including anxiety about treatment or struggles with the impact of a condition on their personal life (e.g. career, family) [16].

Although compassion has a close relationship with empathy [18], and sometimes the two terms are used interchangeably, a distinction can be made between them. Empathy implies experiencing vicariously another’s positive or negative feelings [19]. It is defined as a necessary attribute for compassion, to understand and feel with another [18]. According to Klimecki and Singer [20], this connection can either lead to compassion or to empathetic distress, whereby someone feels overwhelmed and takes strides to withdraw from another’s suffering. The difference between empathy and compassion has been explored recently in neuroscience, which has found particular areas of the brain are activated by these different emotions [21–23]. Preliminary research suggests it may be possible to transform neural networks through activities such as contemplative techniques (e.g. meditation), meaning that compassion may be malleable and augmented through training to increase positive affect, with a compassionate response said to overcome the negative impact of empathic distress [19].

The above overview shows that compassion is not the preserve of healthcare providers. Yet a challenge remains in translating ideals and theories into descriptions and understanding of compassionate care as a process – what it is, how it is delivered, what is required for it to be sustained. Most writing on this topic tends to be descriptive or opinion, rather than empirical work seeking to go beyond the surface to provide a deeper understanding of the topic [24]. A framework has been developed to help HCPs and managers contemplate how to reduce patient suffering, called Compassionate Connected Care [25]. It consists of 4 components: Clinical (e.g. basing care on up-to-date evidence), operational (e.g. good co-ordination of care), cultural (e.g. not being driven by targets and data collection) and behavioural (e.g. respectful interactions, providing complete and understandable information). Whilst useful, it does not appear to have been developed through a ground-up approach, by consulting with those delivering care on a daily basis. A study that did seek to do this, involving one hospital ward in the UK, identified ‘compassionate relationship centred care’ as encompassing “caring conversations that allow the development of knowledge about the person (be it staff member, patient or family member) that provides insights into: who they are and what matters to them, and an understanding about how they feel about their experience” [26]. This work can be built upon to include HCPs from a range of disciplines and to consider different areas of care (e.g. long-term conditions, primary care). Such further research will allow for the development of a theory on compassionate care that is grounded in practice, which can then be used to underpin initiatives aimed at supporting and sustaining positive interactions between patients and HCPs. This paper reports on a study that provides a model for understanding processes associated with compassion in healthcare that can make a contribution to future developments in terms of theory generation and interventions to facilitate compassionate care.

### Aim

Our research set out to explore compassionate care from the perspective of staff working in health settings. It aimed to produce a framework for understanding compassionate care and to investigate:

1. How staff make sense of the concept ‘compassionate care’.
2. Their rationale or motivation for providing compassionate care.
3. Benefits and drawbacks for staff of providing compassionate care.
4. Possible barriers and enablers to compassionate care.

To give a focus to these aims and to provide some homogeneity within the sample, providing care for people with type 2 diabetes was chosen as a critical case for understanding compassionate care. Diabetes is a long-term condition that calls for regular contact with a range of HCPs, meaning there is scope for variation in views about how best to support patients compassionately.

## Methods

**Fig. 1:**
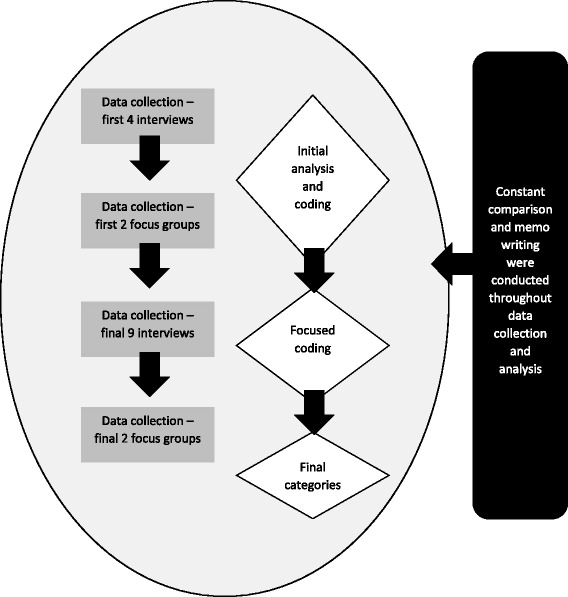
Shows the stages involved in conducting the study. Data collection and analysis occurred in tandem, as is expected in a grounded theory project [28]

A qualitative methodology was appropriate for exploring the objectives listed above. Specifically, constructivist grounded theory was used because it places an emphasis on uncovering contextualised social processes [27] and focuses on what people do and how they do it [28]. We developed an understanding of the topic through concurrent data collection and analysis [29]. The study was approved by the University of Warwick’s Biomedical and Scientific Research Ethics Committee. Stages associated with data collection and analysis are presented in Fig. 1.

### Sample

Initially, purposive sampling was used to access those able to illuminate the topic of interest, namely staff working with patients with type 2 diabetes. Maximum variation was sought in order to involve people who differed in terms of experience, gender and roles (professional and support workers) from primary and secondary care. They were recruited through the following routes, from across the UK: a) advertising at two NHS Trusts (one in the South of England, the other in the Midlands), b) presenting at a diabetes education club at the University of Warwick, c) emailing students undertaking a postgraduate qualification or a certificate in diabetes care at Warwick Medical School, d) Diabetes UK’s newsletter for professionals. As the study continued, snowballing was employed to support theoretical sampling, an approach that allows for further development of categories by involving cases with specific characteristics that help to elucidate a category’s properties [28]. The emerging analysis highlighted a need to seek out professionals who were relatively new to the field, as well as GPs, which we did as the study progressed. All participants received a study information sheet, and gave written or verbally recorded consent to take part and for use of their anonymised quotations when reporting and disseminating findings.

### Data collection

Participants opted to take part in an interview or focus group between May and October 2015. We employed more than one method to develop a broader insight on the topic and to help with checking the credibility of our emerging analysis. Interviewing is common in grounded theory, in which open ended questions are posed to elicit detailed responses [28]. Four interviews were conducted initially and analysed to produce some early concepts, which were then tested in the first two focus groups. The interactive nature of focus groups proved useful in exploring these concepts as participants discussed topics together (e.g. the link between professionalism and compassion). After this we conducted further interviews to explore in greater depth our emerging ideas. Another two focus groups were then carried out to examine these ideas by posing questions to participants to answer collectively. Recruitment stopped at the point of data saturation, when gathering more data did not further understanding of categories or provide additional insights. Interviews and focus groups were digitally, audio-recorded and transcribed verbatim.

Semi-structured interviews gave people time to express themselves and enabled the first author to pursue unanticipated areas raised by participants. A topic guide was developed at the outset that was amended, over time (items removed or added), to reflect incoming data and the emerging analysis. Items on the initial topic guide included: *What comes to mind when I say ‘compassionate care’? How do you feel when you are able to deliver care compassionately? How do you feel when unable to do so?* Questions added as data collection progressed included: *What is it to be a professional? Where does compassion fit into this? When you see someone caring compassionately, what might they be doing or saying?* Most interviews were conducted by telephone; two were face-to-face. They lasted between 40 and 75 min.

Focus groups were useful for exploring collective approaches and attitudes to compassion, and for understanding organisational dynamics. As with interviews, a topic guide was developed. The focus group topic guide contained opening, introductory, main and ending questions; it covered similar items to interviews. Focus groups lasted between 40 and 80 min.

All data were gathered by the first author, who was not known to participants beforehand. She has no clinical background but entered the field as someone with health services research experience and an interest in how HCPs make sense of compassion. Remaining members of the team have a background in research and in academia but also in nursing or general practice.

### Analysis

Interview and focus group data were analysed together, an approach that has been used successfully by others who have included both methods in a grounded theory study [30, 31] Analysis started with line-by-line coding, which took place whilst data collection progressed. These codes were based on gerunds (words ending in ‘ing’ to signify an action or state), to help with comprehending processes associated with compassionate care, whilst staying close to the words of participants [28]. This stage of the analysis facilitated an understanding of what was happening in the data. The first author completed initial coding of all transcripts in pen and paper format. The rest of the team examined transcripts from the first 4 interviews and from 2 focus groups. Discussion of these early codes took place at regular meetings.

Analysis then moved to focused coding, whereby significant or frequent initial codes were considered, to see which made most analytic sense for categorising data [28]. A summary of initial codes from each case was formed (using post-it notes). This enabled the first author to cluster them to develop the focused codes. She shared these with the rest of the team, which allowed for conversations (face-to-face and via email) of alternative ways to cluster codes. Focused coding commenced after 3 focus groups and 10 interviews, when it was felt that key ideas were recurring. After focused codes had been developed, they were entered into the computer programme NVIVO (Version 10) and applied to transcripts of focus groups and interviews. Further data collection did not add to existing focused codes, but helped with clarifying final categories, especially the one we labelled ‘professional compassion’ (see below). Focused codes and their relationship with key categories are presented in Table 1.

**Table 1:**
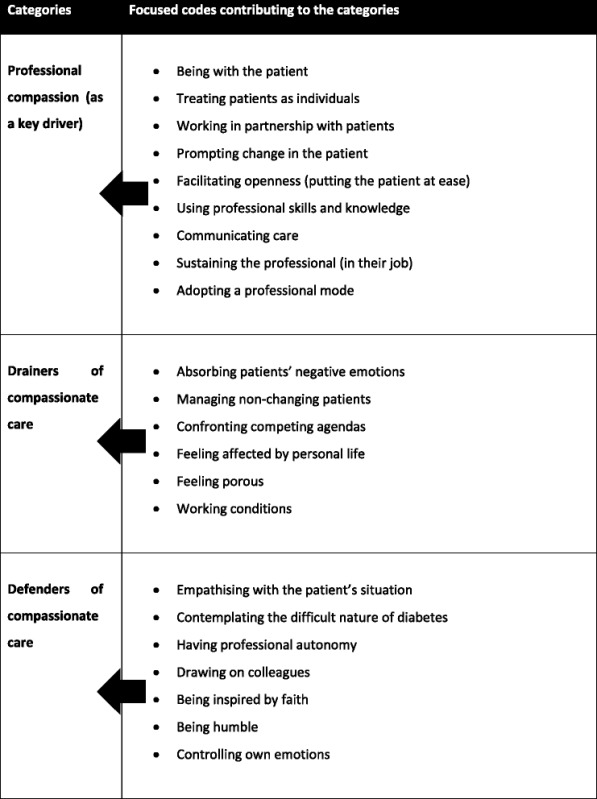
Focused codes were developed following initial analysis, as data collection progressed, and were then clustered into the categories presented in the model (see Fig. 2)

Memos were written to capture analytic processes throughout the study. They present a space for documenting constant comparison, a cornerstone of grounded theory, which entails comparing “data with data, data with codes, codes with codes, codes with categories, and their finished analyses with relevant theoretical and research literature” [32]. Constant comparison was used to compare initial codes when clustering them to be more focused, and then to consider emerging categories within the data. As part of constant comparison, we noted when people raised the same point and explored how what they said was similar or differed. Steps taken to address rigour and trustworthiness within the study are outlined in Table 2.

**Table 2:** Approaches taken towards rigour and trustworthiness

1. The first author documented her thoughts about compassionate care at the start of the project and returned to this description on several occasions to ensure that the study’s progression was not being stifled by these preconceptions.	
2. More than one person, from a range of backgrounds, was involved in the analysis.	
3. An audit trail was kept within NVIVO, documenting decisions made in relation to data collection and analysis.	
4. Regular team meetings allowed questions to be posed of the data. Disagreement was addressed through discussion.	
5. Data were collected from a range of professionals in terms of role, experience and work setting (primary or secondary care).	
6. Emerging concepts from the data were checked out as data collection progressed by posing specific questions to further participants.	

## Results

Data were collected from 36 healthcare staff (29 females, 7 males), with differing amounts of experience in working with patients who had type 2 diabetes (range from one month to 36 years). We achieved variation in the professional groups represented: Nurses (including specialist nurses) = 13; Doctors (including consultants and general practitioners) = 7; Podiatrists = 6; Healthcare assistants/support workers = 5; Dieticians = 3; Administrative staff = 2. Three of the focus groups (the first consisting of 3 doctors, 2 nurses and a podiatrist, the second a mix of 3 nurses and 3 healthcare assistants, and the third composed of 2 nurses and a support worker) involved HCPs based in a hospital. The final one was attended by 8 podiatry team members working in the community, including two of their administrative staff (it was felt relevant to involve them in the discussion because they were the first port of call for patients contacting the service). As for the 13 interviews, most (*n* = 9) were conducted with staff from primary care or working in the community. The rest of the interviewees (*n* = 4) were secondary care employees.

**Fig. 2:**
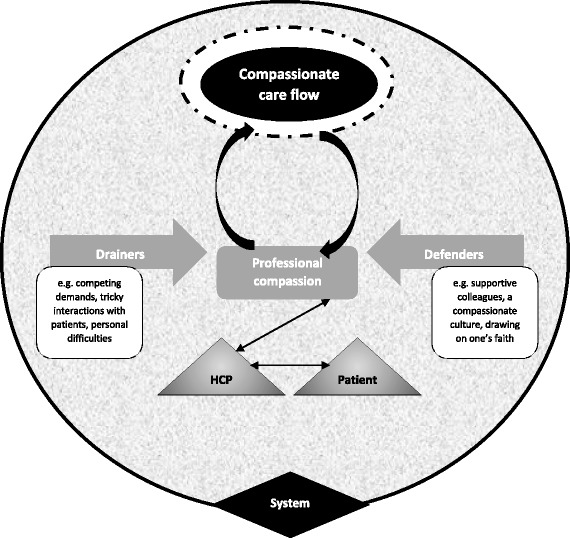
The compassionate care flow. Our model highlights how the flow of compassionate care is shaped by more than an individual HCP’s wish to engage with patients with care and kindness, because it is embedded within a system. Professional compassion drove this flow but was likewise sustained by having the flow of compassionate care validated or displayed (e.g. HCPs being thanked by patients or seeing colleagues caring compassionately). The flow could be punctured or upheld by drainers and defenders respectively, through their impact on professional compassion, which represents an inner desire to improve patient well-being and to act as one would expect from someone in a healthcare role

Throughout the project, the research team discussed a number of topics to arise from the data, such as the meaning of compassionate care in terms of being a professional and how compassionate care ran along a continuum rather than being something that was turned on and off. These discussions resulted in the focused codes listed in Table 1, which were clustered into the key categories that underpin the model developed as an end product of the research (see Fig. 2). It centres on the overarching concept to emerge from the analysis - the flow of compassionate care. This represents an ideal way of being that is associated with the aspirations of HCPs, the expectations of patients and is generated within a healthcare system. Key categories contributing to this overarching concept include professional compassion, which is affected by drainers and defenders. We will describe each of these key categories, and their contribution to the compassionate care flow, along with illustrations from the data, before commenting on the implications of our findings for practice and policy.

### Fuelling the flow through professional compassion

Compassion itself was described by participants as innate and a driver to undertaking a career in healthcare. However, several people used the qualifier ‘professional’ when talking about compassion in the workplace. Professional compassion was said to be exhibited by good communication, being alert to patients’ needs and small acts of kindness. It appeared to be part of participants’ job-related repertoire, alongside medical skills and knowledge. As a workplace experience, professional compassion was described as involving some degree of learning from peers (e.g. watching how others behaved, discussing clinical encounters informally or in supervision). It was depicted as functional, being used to improve people’s health by helping staff to connect with patients to build a rapport with them in order to promote good self-care. Similarly, it was painted as a tool that enabled HCPs to be patient-centred:

FG2 P2: “You can be professional and kind of just get through it and then compassion kind of makes you an individual, it makes that person feel like they are an individual, so the care’s for them directly…”

Int 3: “I think it’s just trying to understand where that person is at the moment and what they are physically capable of and just saying to them…in the ideal world this, this and this should happen but actually today we’ll concentrate on this one element. It’s about developing that relationship.”

Sometimes participants provided what was described as “tough love” (FG4 P5), including the use of “scare tactics” (FG4 P3) (e.g. emphasising what medical problems might befall patients). Adopting such an approach was still seen as professional compassion by most because it was driven by a wish to prevent future medical problems. This highlights that although professional compassion could be spontaneous, occasionally it was a more conscious encounter, requiring effort:

Int 4: “Some days you have to physically think about doing it [compassionate care]. Most of the time I would suggest, or I hope that it just comes naturally…but I think tough times maybe you have to think to yourself OK…those groups of patients that very much have done it to themselves, through their lack of own self-care. It’s about supporting them through that realisation because they do get it in the end but think damn, I’ve done this to myself and then people get angry and I think it’s really important then to sort of show them…that’s where real compassion comes.”

In sum, professional compassion represented a work-based standard. It was something that individuals employed to facilitate their interaction with patients and to improve people’s health. It prompted them to do their best for a patient and, in that sense, energised the compassionate care flow. Professional compassion was likewise sustained by sensing that compassionate care had been delivered by them (e.g. when a patient thanked them for their work) or by their colleagues, suggesting that a feedback loop was at play (see Fig. 2):

Int 5: “I feel appreciative at work, if a colleague…gives me good feedback about a patient who may have given good feedback about me to them…I’ve felt satisfied from a professional point of view…it makes you appreciate that actually you try to do a good job and I think that receiving compassion enables you and it motivates you more to give compassion back…”

This feedback loop could be fractured by a range of factors that made individuals question whether what they were doing was worthwhile or sustainable. As outlined in the next section, if they felt overwhelmed and unable to contain their anxieties or frustrations, it threatened to damage the compassionate care flow.

### Drainers puncturing the flow of compassionate care

The flow of compassionate care could be affected by the presence of what we have defined as drainers, which stifled the ability to express professional compassion. They came in a range of guises. For example, although eliciting and addressing patients’ needs was alluded to when participants discussed compassionate care, this could be impeded by a lack of rapport; when patients were perceived as dismissive or demanding or overly dependent on HCPs, there was a sense of compassionate care depleting:

FG3 P1: “We get people coming in and shouting at us. Not often, not often, but we do and that can be quite hard and it’s quite hard to feel compassionate to someone when they’re yelling at you…”

Compassionate care was similarly said to diminish when faced with someone who did not alter their health behaviours and engage in self-care. Interactions with such patients did not replenish the compassionate care flow because staff felt a lack of positive feedback from doing a good job; instead, they could believe they were failing in their professional role:

Int 1: “If someone comes in saying I’m not really interested in any of this, I don’t need it then the passion goes out and I, I sort of like do the minimum…I remember vividly wanting to walk away from nursing…because I couldn’t get it right. So I really felt that I was failing people… I think this is actually quite key in terms of compassionate care because you can deliver, you keep giving and then sometimes you don’t see the results, so you can’t see success…”

Int 2: “… [you feel] you’ve not been effective in being able to convince them to change things to prevent a diabetic complication that’s irreversible…personally it’s a difficulty sometimes because I feel I have personally failed…”

The system within which participants worked could be another drain on their expression of professional compassion and, therefore, the flow. Time pressures in particular (e.g. only having 10 min appointments) left HCPs feeling unable to connect with a patient because of insufficient opportunities to sit back, listen and reflect on what someone was saying. This lack of time was compounded by the following competing demands that diverted energy away from activities that contributed to compassionate care: increasing caseloads, lack of privacy to discuss things on a ward, chasing targets, completing paperwork/logging data on a computer, being short staffed, fear of litigation. The following data extract illustrates this point:

FG4 P1: “I think you’re expected to do more and more in your time slot. You have thrown at you all the time about litigation and your notes…you’re there ultimately for patient care and it can be really frustrating that everything else gets in the way, having to put your mileage on and sort it…

FG4 P8: …time constraints and various other pressures, it doesn’t alter your level of compassion. It just, it might be squeezed a little bit. It’s a bit squeezed.

FG4 P5: The more they put on us, the less we’ve got to give out.”

Working under such competing demands could mean the patient’s agenda was lost according to participants, blocking the compassionate care flow by jeopardising professional compassion in terms of being patient-centred. There was also a danger of HCPs becoming desensitised to others’ distress and executing tasks by rote. One participant (Int 11) noted being pulled in two directions – having to tick boxes and audit her work whilst bearing in mind that for the patient, receiving and living with a diagnosis of diabetes was life changing. Another participant suggested that this split started early in her professional career:

Int 12: “…medical school punctures the compassion out of you …I remember my first two years I did 74 exams in various different subjects…you get tired and then, you don’t talk to people out of intrigue of their life, you talk to people to get a history, to get a diagnosis…Actually we’re talking about people and illnesses and that’s not stressed at med school.”

A further factor that could impede HCPs’ ability to focus on the person in front of them was the presence of personal stressors (e.g. family illness, problems in their relationship), although one participant was clear that this should not interfere with how a patient was treated:

Int 4: “…it’s difficult to come to work if you’ve had things happen at home. You might not always feel very kind…I’d just ignore the personal life until I got home. If I couldn’t do it I wouldn’t come to work…it’s not their fault something might not be OK somewhere else.”

### Defenders of the compassionate care flow

In contrast to drainers, defenders reinforced the compassionate care flow by upholding professional compassion (see Fig. 2). Defenders included acknowledging the reality of patients’ circumstances (e.g. diabetes as a lifelong, difficult condition to regulate) and being curious about someone’s behaviour in order to connect and be with that individual rather than taking a defensive or detached stance. It might call on HCPs to suppress negative emotions:

FG2 P3: “…patients are all different and some you will just bite your lips with because…they are here, treating us like servants…”

Int 7: “I might have seen a lady in clinic and she hasn’t really got that much to worry about but to her it is such a big deal and it’s very hard cause I feel like I have to bite my tongue cause if I look at what I see other people going through and they’re coping, it’s very hard not to say or not to feel frustrated…”

This seemed easier to do with more experience in the role and participants noted that over time their view of what constituted compassionate care was affected by a better understanding of patients as people:

FG1 P1: “I think respect for patients. Treat them as people rather than diseases is one thing because you can get carried away with this type 2 diabetes, hypertension, obesity and you start treating that rather than the person.”

Int 2: “I’ve come to recognise we can make small strides and where we make them we should celebrate but also recognise that two steps back doesn’t mean it’s going to be 3 and 4 steps back, that actually we can, that might just be a temporary blip and then finding what motivates that person onwards.”

Data indicated that personally identifying with a patient’s situation could bolster the flow of compassionate care by supporting professional compassion through focusing on the individual’s needs. This might involve HCPs contemplating how they would want a loved one to be treated and admitting that they also found adopting healthy behaviours difficult. It was said to be helpful to reflect on one’s limitations to engender change in patients and not be self-critical. This ability to avoid blaming oneself seemed to be another skill that developed with experience; when first qualified, participants recalled expecting to make a difference to the health of each person they cared for, but became more pragmatic overtime. A couple of HCPs did talk about deciding to perceive patients who seemed to lack motivation to self-care as a welcomed challenge, which enabled them to continue providing professional compassion:

FG4 P1: “…there’s been the odd few where I’ve thought you are difficult, I’m struggling with you but in the end you really, cause when you do get them to open up you understand why they can be difficult. Some of the like cantankerous old men can really become my favourites.”

Autonomy in the workplace facilitated compassionate care according to several participants; it let them plan their own timetable and extend their contact with a patient, if required, to develop a rapport that was linked to professional compassion. Likewise, drawing on supportive colleagues helped with upholding the flow because when individuals felt they had engaged in a difficult consultation, they were able to vent with team members or could seek their advice on how to proceed. If a clinical interaction was becoming too fractious or unproductive, the patient could be transferred to a colleague rather than risking the flow of compassionate care collapsing. For this support to be realised, participants emphasised the importance of belonging to a team that saw the humanity of staff and showed compassion towards providers of care. In this respect, work culture was noted as assisting with the compassionate care flow by having positive role models and a supportive organisation:

FG1 P6: “…if everybody around you is compassionate, it’s natural to be compassionate, whereas if everybody around you isn’t then it’s quite difficult to be the only one in that group.”

Int 8: “…[the Trust] needs to think about duty of care to staff because…it can impact on the clinician because they can’t do a good job, they’re not achieving results they should be achieving…So there does need to be some compassion there and understanding.”

Three participants talked about being inspired by their faith to remain compassionate in the face of drainers outlined above, seeing compassion as fundamental to their religious beliefs. In addition, being humble could buttress the flow of compassionate care. This was described as providing compassion as a professional, with no expectation of anything in return. Accepting that lapses in practice could occur and striving to put these right was another example:

Int 5: “…I was getting a call from reception saying a patient’s kind of kicking off, threatening to sue you…because you were running late and I was like OK, so when the patient came in I just greeted the patient, he had a few words to say and I apologised, explained to him the situation and then his whole tone, the whole tone of the situation just changed and actually he left the consultation shaking my hand and thanking me, happy, positive.”

## Discussion

We have described a novel model based on flow (see Fig. 2) that recognises how compassionate care is provided within healthcare settings along a continuum and can be affected by a complex range of interpersonal and organisational defenders and drainers. These influenced professional compassion, which appeared to energise the compassionate care flow and was driven by an innate desire to help others, anchored around work-related goals and expectations. Our model highlights that although compassion is commonly related to an individual, when applied to healthcare it is social in nature, given and received in a specific setting, and shaped by being part of a professional community. This challenges policy on compassionate care that focuses narrowly around an individual’s behaviours, a criticism raised by Crawford and colleagues [33].

Flow, used to describe an ideal in terms of compassionate care, is a concept linked with positive psychology [34] to define an activity that provides inherent pleasure or satisfaction, whereby someone becomes completely absorbed [35]. It is said to occur as an optimal experience, involving “a fine balance between one’s ability to act and the available opportunities for action” [36], that is “so engrossing and enjoyable that the activity becomes worth doing for its own sake without the impetus of extrinsic motivation” [37]. This is similar, in some respects, to the compassionate care flow in that individuals talked about being motivated to experience this state because of how it made them feel. However, sometimes this intrinsic reward could be compromised by drainers depicted in Fig. 2, meaning that external reinforcement was also important (e.g. support from colleagues, positive feedback from patients). Participants did mention how compassionate care was, at times, imbedded in their work, but it could be consciously contemplated when something challenged its expression. In that sense, it relates to the psychological notion of flow, which can be lost when attention shifts from the activity in hand [38]. Both psychological flow and the model we present in relation to compassionate care emphasise the importance of how work is designed, with people engaged in activities where they have autonomy and variety of tasks more likely to experience flow [35, 37, 38].

Use of the term professional as a prefix when describing compassion in the workplace is a new way of considering this topic. It highlights the specific nature of compassionate care in terms of the contribution it makes to a patient’s health. A focus on symptom relief/avoidance contributed to the delivery of ‘tough love’ (being stern with the aim of alleviating future health problems), an approach noted by other authors [39]. There was also a sense of professional compassion as something that was anticipated within a healthcare setting. However, as suggested in our data, HCPs may feel drained of compassion when working in a culture of threat that is replete with discourses of insecurity, targets, low staff levels and time pressures [33]. Feeling overwhelmed by competing external demands may contribute to compassion fatigue, a form of psychological exhaustion that can occur due to contextual factors [40], such as employees believing that an organisation does not care about them as a person. It is argued that more attention should be given to the needs and welfare of staff because “without a degree of reciprocity, without the carer’s needs being recognised, and without finding meaning in the interaction, one-way compassion is likely to lead to burnout” [41]. This has been referred to as bio-directional compassion [33], which is similar to the feedback loop represented in Fig. 2.

It was interesting to note that although an emphasis has been placed on suffering as part of compassion [18], this term was not used by HCPs involved in our research. It could be argued that suffering was alluded to when they mentioned addressing someone’s physical struggles and being attentive when patients broached sensitive topics. Alternatively, it may be that participants did not see people with diabetes as ‘suffering’ because this term has been depicted by HCPs as too vague or too burdensome when describing what they do in their work [42]. Yet suffering can come from being part of a healthcare system, if it causes patients to feel confused and anxious (e.g. due to delays, poor communication, lack of coordinated care) [43]. Addressing compassionate care at an organisational level so a culture supports staff in providing compassionate care is thus important.

The relational aspect of compassion described in the existing literature [5, 8, 9] did surface within our data. It risked being curtailed by the nature of healthcare environments (e.g. competing demands, lack of time, short staff), resulting in empathic distress [20], whereby professional compassion meant HCPs had a desire to deliver compassionate care but were at times stifled by an unsupportive work environment.

### Strengths and limitations

Our study used approaches listed in Table 2 to support the robust generation and interpretation of data. For example, more than one person was involved in the analysis, which used principles associated with grounded theory to develop a model based on HCPs’ understanding of compassionate care. It should be noted that data were collected from a diverse group of 36 participants but discussion focused on the care of patients with a specific condition. Nevertheless, we believe that findings have wider application because most of the drainers and defenders are likely to be experienced by staff from other fields, especially those working with people who have long-term conditions. Data were collected from individuals volunteering to be part of an interview or focus group who may have had a particular interest in this topic. In addition, they were recounting their perceptions of what they did or saw in practice, which may be different from what actually occurred. That said, we were interested in understanding the meaning of compassionate care from their perspective and they did provide a range of views on the topic, which enabled us to generate the model presented above.

### Clinical and research implications

Several challenges to enacting compassionate care in practice were identified in our study. The model we produced provides a means for thinking about these that could be used by HCPs to reflect on their own practice, and by healthcare leaders to evaluate what is happening at an organisational level. Using the ideas presented in our model, future research might explore whether the compassionate care flow can be sustained against drainers by compassion training to avoid empathic distress [23]. It could also be employed to develop and evaluate practice environments that aim to augment defenders of the compassionate care flow.

Further work is now needed to consolidate and refine our ideas. For example, exploring whether our model extends to non-health related settings where compassion may be expected, such as the clergy, social or youth work, and teaching, in which different situational and interactional defenders and drainers may be present. Furthermore, we could develop and test the model further by collecting data from patients; their perspectives may confirm the model or suggest the need for its modification. For example, research could determine how patients make sense of chronic conditions (like diabetes) within their daily lives to find better ways of working with HCPs, given that the issue of challenging patient-professional interactions was raised as a threat to the compassionate care flow. This links to the relational aspect of compassionate care. ‘Being with’ patients [44], by connecting on a human level, requires interpersonal competence [45] and is a skilled activity (e.g. understanding how to address the patient’s particular situation, taking into account his or her individual needs) that should be recognised and valued. However, promoting relational aspects of care in a culture judging performance on quantitative measures relating to efficiency and throughput can be difficult [45].

## Conclusion

Data from staff working with patients who have type 2 diabetes was used to develop a novel model of compassionate care centred on the idea of flow. It was underpinned by the categories of professional compassion, drainers and defenders, and emphasises how compassionate care is broader than a personal value. Participants made sense of compassionate care as present within a particular setting, depicting it as something that evolved and was learnt through working in healthcare, and shaped by the influence of colleagues, patients and organisational demands and expectations. They were motivated to provide compassionate care because it was seen as important for developing a rapport with patients and played a role in improving people’s health. Benefits were positive interactions and job satisfaction, but competing demands in terms of time and resources could make compassionate care difficult, meaning it became something that had to be thought about rather than just being innate. Findings highlight the importance of attending to environmental factors that can augment defenders and address drainers of the compassionate care flow.

## Abbreviations

HCP: Healthcare professional
